# Differential binding patterns of anti-sulfatide antibodies to glial membranes

**DOI:** 10.1016/j.jneuroim.2018.07.004

**Published:** 2018-10-15

**Authors:** Gavin R. Meehan, Rhona McGonigal, Madeleine E. Cunningham, Yuzhong Wang, Jennifer A. Barrie, Susan K. Halstead, Dawn Gourlay, Denggao Yao, Hugh J. Willison

**Affiliations:** Neuroimmunology Group, Institute of Infection, Immunity and Inflammation, College of Medical, Veterinary and Life Sciences, University of Glasgow, UK

**Keywords:** Sulfatide, Monoclonal antibody, Neuropathy, Complement, Myelin, GBS, Guillain-Barré syndrome, MS, multiple sclerosis, CST, cerebroside sulfotransferase, MAC, membrane attack complex, Sulf, sulfatide, Chol, cholesterol, DCP, dicetyl phosphate, SM, sphingomyelin, GalC, galactosyl ceramide, WLE, whole lipid extract, BSA, bovine serum albumin, NGS, normal goat serum, CNS, central nervous system, BNB, blood-nerve barrier

## Abstract

Sulfatide is a major glycosphingolipid in myelin and a target for autoantibodies in autoimmune neuropathies. However neuropathy disease models have not been widely established, in part because currently available monoclonal antibodies to sulfatide may not represent the diversity of anti-sulfatide antibody binding patterns found in neuropathy patients. We sought to address this issue by generating and characterising a panel of new anti-sulfatide monoclonal antibodies. These antibodies have sulfatide reactivity distinct from existing antibodies in assays and in binding to peripheral nerve tissues and can be used to provide insights into the pathophysiological roles of anti-sulfatide antibodies in demyelinating neuropathies.

## Highlights

• Sulfatide liposomes generate anti-sulfatide antibodies in mice.
• Anti-sulfatide antibodies have diverse binding patterns both *in vitro* and *in vivo.*
• Following passive immunisation, prominent binding is seen in peripheral nerve distal myelinated fibre paranodal membranes.
• Antibodies are capable of binding and injuring the myelin of the distal motor nerve.

## Graphical abstract

**Figure:**
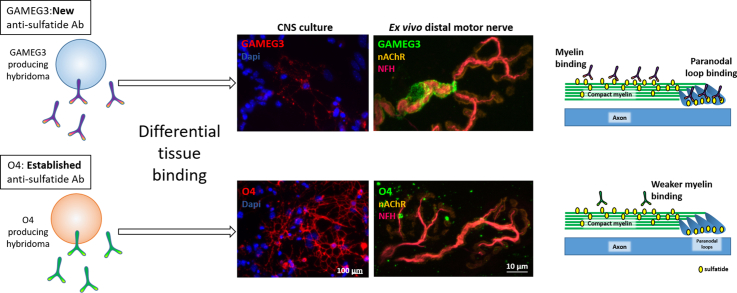

## 1 Introduction

Sulfoglycolipids are common components of the mammalian plasma membrane that are distinguished by the presence of a sulfate group. Among the more abundant sulfoglycolipids is sulfatide, 3-O sulfogalactosylceramide, formed from galactocerebroside through the enzymatic action of cerebroside sulfotransferase (CST)(Honke et al., 2002). Sulfatide is expressed at low levels in many tissues but is particularly enriched in the myelin sheaths of the central and peripheral nervous systems (Takahashi and Suzuki, 2012).

In the nervous system sulfatide modulates diverse functions including myelin maintenance and stabilisation (Hirahara et al., 2004; Palavicini et al., 2016). Its importance in myelin homeostasis is evidenced in sulfatide-deficient mice lacking cerebroside sulfotransferase (CST^−/−^) which develop normally up to six weeks, thereafter displaying progressive hindlimb paralysis, tremor and ataxia (Honke et al., 2002). Morphologically, myelinated nerves from CST^−/−^ mice initially form compact myelin and exhibit normal axons and ion channel clustering at the nodes of Ranvier. After 6 weeks, disorganisation of the myelin and disassembly of the nodes of Ranvier and paranodal junctions occurs, indicated by decreased Na^+^ and K^+^ channel clustering and abnormal distribution of K^+^ channels that are misplaced from the juxtaparanodes into the paranode (Hoshi et al., 2007; Ishibashi et al., 2002; Marcus et al., 2006; Takano et al., 2012). These disturbances directly correlate with sulfatide content in the peripheral nerve, as evidenced by combined biochemical and functional studies (Hayashi et al., 2013). However, the temporal and mechanistic sequence by which these demyelinating events evolve is not understood, in part owing to limited information on sulfatide distribution in glial cell membranes.

In addition to its essential role in myelin homeostasis, sulfatide has also long been considered as a possible autoantigen in disease in which antibody targeting of the glycolipid in myelin would be expected to have detrimental effect on nerve function. Antibodies that bind sulfatide are widespread in both normal and disease populations but they are particularly prevalent in patients with multiple sclerosis (MS), Guillain-Barré syndrome (GBS) and paraproteinaemia-associated neuropathy (Nobile-Orazio et al., 2014; Nobile-Orazio et al., 1994; van den Berg et al., 1993). In MS, anti-sulfatide antibodies have been found in cerebrospinal fluid, suggesting local intrathecal synthesis by ectopic B cells (Brennan et al., 2011; Ilyas et al., 2003; Kanter et al., 2006). In cohorts of GBS and other autoimmune neuropathy cases, serum anti-sulfatide antibodies have been reported with varying sensitivity and specificity (Fredman et al., 1991; Halstead et al., 2016; Ilyas et al., 1991; Morikawa et al., 2016; Petratos et al., 1999; Rinaldi et al., 2013; Terryberry et al., 1995). Antibodies binding to sulfatide have been predominantly linked with demyelinating neuropathies, consistent with the abundance of the glycolipid in myelin, but have also been associated with neuropathies characterised by prominent axonal loss (Carpo et al., 2000). Overall, evidence indirectly indicates that anti-sulfatide antibodies are associated with a proportion of autoimmune neuropathy cases and may play a role in pathogenesis; however experimental evidence on causality is lacking and their use as a diagnostic or prognostic factor is still uncertain (Giannotta et al., 2015).

Attempts to study and model demyelinating neuropathies have primarily been carried out using monoclonal antibodies. The most widely used anti-sulfatide monoclonal antibody is the IgM antibody termed O4 (Sommer and Schachner, 1981), despite the generation and characterisation of other antibodies (Cheng et al., 2005; Colsch et al., 2008; Fredman et al., 1988; Hofstetter et al., 1984). Using O4, the demyelinating and dysmyelinating effects of anti-sulfatide antibodies *in vitro* and *in vivo* have been studied in the CNS (Elliott et al., 2012; Kanter et al., 2006; Rosenbluth and Moon, 2003; Rosenbluth et al., 2003); however, similar studies examining the effects of anti-sulfatide antibodies in the PNS have been very limited. One experimental model of peripheral nerve demyelination has been induced through sulfatide immunisation, resulting in neuropathy accompanied by IgG anti-sulfatide antibodies (Qin and Guan, 1997). As antibodies of the IgG isotype can opsonise tissue, they are capable of eliciting a pathogenic response through activation of complement. Complement deposits have been observed in human nerve biopsies from patients with circulating anti-lipid antibodies, and complement-mediated injury is a recognised mechanism of pathology in demyelinating diseases (Ferrari et al., 1998; Hafer-Macko et al., 1996; Storch and Lassmann, 1997).

Autoimmune neuropathy models within our own laboratory using O4 have had limited success, in part due to difficulty in clearly demonstrating binding of the O4 antibody in live peripheral nerve and nerve-muscle preparations by immunohistology. A previous study showed that human recombinant anti-sulfatide antibodies derived from the cerebrospinal fluid of MS patients were unable to bind live CNS myelin or cells from the oligodendrocyte lineage despite binding sulfatide in solid phase assays (Brennan et al., 2011). It has thus been hypothesised that there are differences in the abilities of antibodies to bind sulfatide in neural plasma membranes, most likely due to steric hindrance in the plane of the plasma membrane preventing antibody access, as previously described for anti-ganglioside antibodies (Greenshields et al., 2009). Furthermore, it is equally possible that the topographical organisation of sulfatide may differ between CNS and PNS. Therefore, to aid in development of models of peripheral demyelination, we isolated a more diverse range of anti-sulfatide antibodies than currently available. Herein, we describe the generation and initial characterisation of the nerve binding properties of a set of IgM and IgG anti-sulfatide monoclonal antibodies for use in investigating pathogenic roles of anti-sulfatide antibodies in models of autoimmune demyelinating neuropathy.

## 2 Methods

### 2.1 Animals

DBA mice were supplied by Charles River, Elpinstone, UK. Mice lacking a functional CST gene (CST^−/−^) and wild type homozygous (CST^+/+^) mice were generated and genotyped as previously described (Honke et al., 2002); the colony was obtained from Karsten Buschard, Bartholin Institute, Copenhagen, Denmark. All mice were housed under controlled conditions consisting of 12 h light/dark cycles in temperature controlled rooms with food and water provided *ad libitum*. All procedures were conducted in accordance with the United Kingdom Animals (Scientific Procedures) Act of 1986.

### 2.2 Active immunisations

Liposomes were generated as described previously (Bowes et al., 2002). They consisted of 100 μg of sulfatide or 500 μg whole lipid extract (WLE) derived from homogenised cauda equina with cholesterol (chol), sphingomyelin (SM) and dicetyl phosphate (DCP) in a 1:5:4:1 M ratio. Mice were initially injected intraperitoneally (IP) with 100 μl of 0.6 mg/ml ovalbumin in 2% aluminium hydroxide on Day 0. They then received further IP injections of 100 μl of liposomes on Day 7, 14 and 21. These were followed by 50 μl intravenous injections of liposomes at 200 μg/ml on days 25, 26 and 27. Blood samples (100 μl) were collected once a week *via* tail venesection, clotted at room temperature for 30 min then centrifuged at 21,000 ×*g* for 20 min at 4 °C.

### 2.3 Hybridoma production

All mice were culled on day 28 post immunisation with a rising concentration of CO_2_ as per UK Home Office guidelines. Spleens were fused with the myeloma cell line P3X63Ag8.653 to create hybridomas, as described previously with minor modifications (Goodyear et al., 1999). Briefly, feeder cells were replaced with hybridoma supplements, either 10% Opticlone (Santa Ana, CA, USA) or 5% HyMax (Antibody Research Corporation, St Charles, MO, USA). Hydridoma supernatants were screened using a lipid microarray instead of ELISA, as described in Section 2.4. This allowed for the simultaneous detection of different antibody isotypes. IgM supernatant was concentrated using the Vivacell 250 (Sartorius, Göttingen, Germany) and quantified using an ELISA kit (Bethyl Laboratories, Montgomery, TX, USA). IgG antibodies were purified using HiTrap protein G affinity purification columns (GE Healthcare, Little Chalfont, UK). These antibodies were quantified using both a Nanodrop 1000 spectrophotometer (ThermoScientific, Waltham, MA, USA) at a wavelength of 280 nm and a BCA protein assay (ThermoScientific, Waltham, MA, USA). IgG subclasses were determined by ELISA using specific anti-mouse antibodies as described previously (Willison and Veitch, 1994). O4 monoclonal antibody, derived from hybridoma supernatant, was kindly gifted by Prof Susan Barnett at the University of Glasgow.

### 2.4 Glycolipid antibody screening by microarray

Serum samples, hybridoma supernatants and purified antibodies were screened against single glycolipids and glycolipid complexes printed using a glycolipid microarray (Halstead et al., 2016). This is a miniaturised version of the combinatorial glycoarray described previously (Rinaldi et al., 2009). Briefly, stock solutions of glycolipids including GM1, GM3, GD1a, GD1b, GT1a, GQ1b, GD3, SGPG, and LM1 were prepared in methanol at 0.4 mg/ml. Glycolipid complexes were prepared by adding an equivalent quantity of each working solution in 1:1 (mol:mol or weight:weight) ratios. For hybridoma screening studies, cholesterol (chol) was made up at a molar weight five times that of all other glycolipids. A microarray printer (sciFLEXARRAYER S3, Scienion, Berlin, Germany) was used to print glycolipid spots in a predefined pattern onto low fluorescence PVDF membrane-covered slides (Millipore, Billerica, MA, USA). Slides were then blocked with 2% BSA/PBS at room temperature for 1 h. Serum samples (100 μl, 1:50 dilution in 1% BSA/PBS), purified anti-sulfatide antibody (10 μg/ml of IgM or 1 μg/ml of IgG) or neat hybridoma supernatants were applied to the slides for 1 h at 4 °C, then washed twice for 15 min in 1% BSA/PBS. AlexaFluor 555 or AlexaFluor 647 conjugated mouse IgM or IgG heavy chain specific antibodies (Jackson ImmunoResearch laboratories, inc, West Grove, PA, USA) were applied to the slides at 2 μg/ml in 1% BSA/PBS for 1 h at 4 °C. Slides were washed 2 × 30 min in 1% BSA/PBS, 2 × 5 min in PBS then finally 5 min in dH_2_O. Slides were imaged using either a Sensovation FLAIR scanner (Sensovation, Radolfzell, Germany) or a GenePix 4300A scanner (Molecular Devices, Sunnyvale, USA). Antibody binding was quantified using the accompanying software with values expressed as median fluorescent intensity (MFI).

### 2.5 Cell culture

Mouse central nervous system (CNS) myelinating cultures were grown on poly-l-lysine coated coverslips as per established methods (Thomson et al., 2008).

### 2.6 Antibody localisation

#### 2.6.1 Myelinating cell cultures

At 28 days *in vitro* (DIV), cell cultures were incubated with anti-sulfatide antibody (10 μg/ml) for 1.5 h at room temperature, rinsed in PBS, fixed with 4% paraformaldehyde (PFA) and permeabilised in −20 °C ethanol for 10 mins. Anti-myelin basic protein (MBP) antibody (#aa82-87 BioRad, 1:500) was applied overnight at 4 °C. After washing in PBS, goat anti-rat Alexafluor 488 and an IgG or IgM goat anti-mouse 555 (Invitrogen, Paisley) secondary antibody was then applied for 30 min at room temperature. The cells were washed again and mounted in Citifluor antifade containing DAPI (Electron Microscopy Sciences Pennsylvania US).

#### 2.6.2 Tissue

Phrenic nerve-diaphragm nerve-muscle preparations were dissected from 6 to 10 week old CST^+/+^ and CST^−/−^ mice, snap frozen, and sectioned transversely at 10 μm. Sections were blocked in 3% NGS in PBS for 1 h at 4 °C, followed by application of the anti-sulfatide antibodies at 10 μg/ml and 1/1500 mouse anti-phosphorylated neurofilament-H antibody (NF-H, #801602 clone SMI31, BioLegend 1:2000) O/N at 4 °C. Slides were washed with PBS followed by incubation with FITC-conjugated goat anti-mouse IgG3 (Fcγ) or IgM (μ) (Southern Biotech, Birmingham, AL, USA) and TRITC-conjugated anti-mouse IgG1 (Fcγ) at 3.33 μg/ml for 1 h at 4 °C in PBS. The slides were washed in PBS and mounted in Citifluor AF1 (Citifluor, Leicester, UK). Whole-mount triangularis sterni (TS) nerve muscle preparations were used for labelling of intact distal myelinated motor nerve bundles. The TS muscle was removed and maintained alive in an oxygenated Ringer's solution (116 mM NaCl, 4.5 mM KCl, 1 mM MgCl2, 2 mM CaCl2, 1 mM NaH2PO4, 23 mM NaHCO3, 11 mM glucose, pH 7.4). TS was treated with 100 μg/ml of either anti-sulfatide IgG3 antibody (GAME-G3) or anti-sulfatide IgM antibody (O4) for 4 h at 32 °C. Control tissue was treated with PBS only. TS were then washed with Ringer's solution and fixed in 4% PFA for 20 min followed by 10 min in 0.1 M glycine to quench unreactive aldehyde groups. Tissue was incubated with 100% EtOH for 10 min at −20 °C, thoroughly washed in PBS and incubated overnight at 4 °C with 1:1500 anti-neurofilament antibody (SMI-31) in 0.3% Triton X-100 + 3% NGS. The TS was washed in PBS and incubated with α-bungarotoxin (BTx; 2 μg/ml; Molecular probes), anti-mouse IgG3/M and anti-mouse IgG1 Alexa Fluor conjugated antibodies (2 μg/ml; Molecular Probes) for 3 h at room temperature. Tissue was rinsed in PBS and mounted in Citifluor.

#### 2.6.3 *In vivo* studies

GAME-G3 antibody (1 mg) was administered to CST^+/+^ and CST^−/−^ mice by IP injection. Mice were sacrificed 20 h later. Diaphragm tissue was immediately removed and snap-frozen. TS was removed for preparation in Ringer's, as above. Lumbrical muscles, sciatic nerve and spinal roots were removed following perfusion with 4% PFA. Whole-mount TS (30 min), lumbricals (1 h), roots (1 h) and sciatic nerve (1 h) were post-fixed at 4 °C. Tissue was stained as above for TS, but omitting the primary anti-sulfatide antibody step and in some cases replacing SMI31 antibody with rat anti-MBP antibody.

### 2.7 Complement activation

Complement activation was performed using a modified whole mount staining protocol. Briefly, TS were treated with 100 μg/ml anti-sulfatide IgG3 antibody (GAME-G3) for 4 h at 32 °C with a source of complement (normal human serum, NHS, 40% in Ringer's). Control tissue was exposed to antibody solution alone. TS were fixed and stained as before with the inclusion of 1 μg/ml mouse anti-human C5b-9 (MAC) antibody (1/50, Dako, Santa Clara, CA, USA) overnight. Tissue was washed in PBS and incubated in isotype-specific Alexa Fluor 488-, 555- and 647- conjugated antibodies (2 μg/ml; Molecular Probes) for 3 h at room temperature. Tissue was rinsed in PBS and mounted in Citifluor.

### 2.8 Antibody internalisation

Baseline blood samples (day −1) were taken from mice *via* tail vein venesection one day prior to an IP injection of 250 μg of GAME-G3 (day 0). Further blood samples were taken on days 1, 3 and 6 and a terminal sample was taken on day 7. These were processed as per Section 2.2. The serum was screened for anti-sulfatide antibodies by ELISA using the previously described method (Cunningham et al., 2016).

### 2.9 Statistical analysis

All graphs and statistical analyses were produced using GraphPad Prism 6 (GraphPad Software Inc., San Diego, CA, USA). *P* values < 0.05 were deemed to be significant.

### 2.10 Microscopy

Representative images were taken using an epifluorescent Axio Imager Z1 microscope with ApoTome attachment (Carl Zeiss, Oberkochen, Germany).

## 3 Results

### 3.1 Anti-sulfatide antibody serum response in different mouse strains

**Fig. 1:**
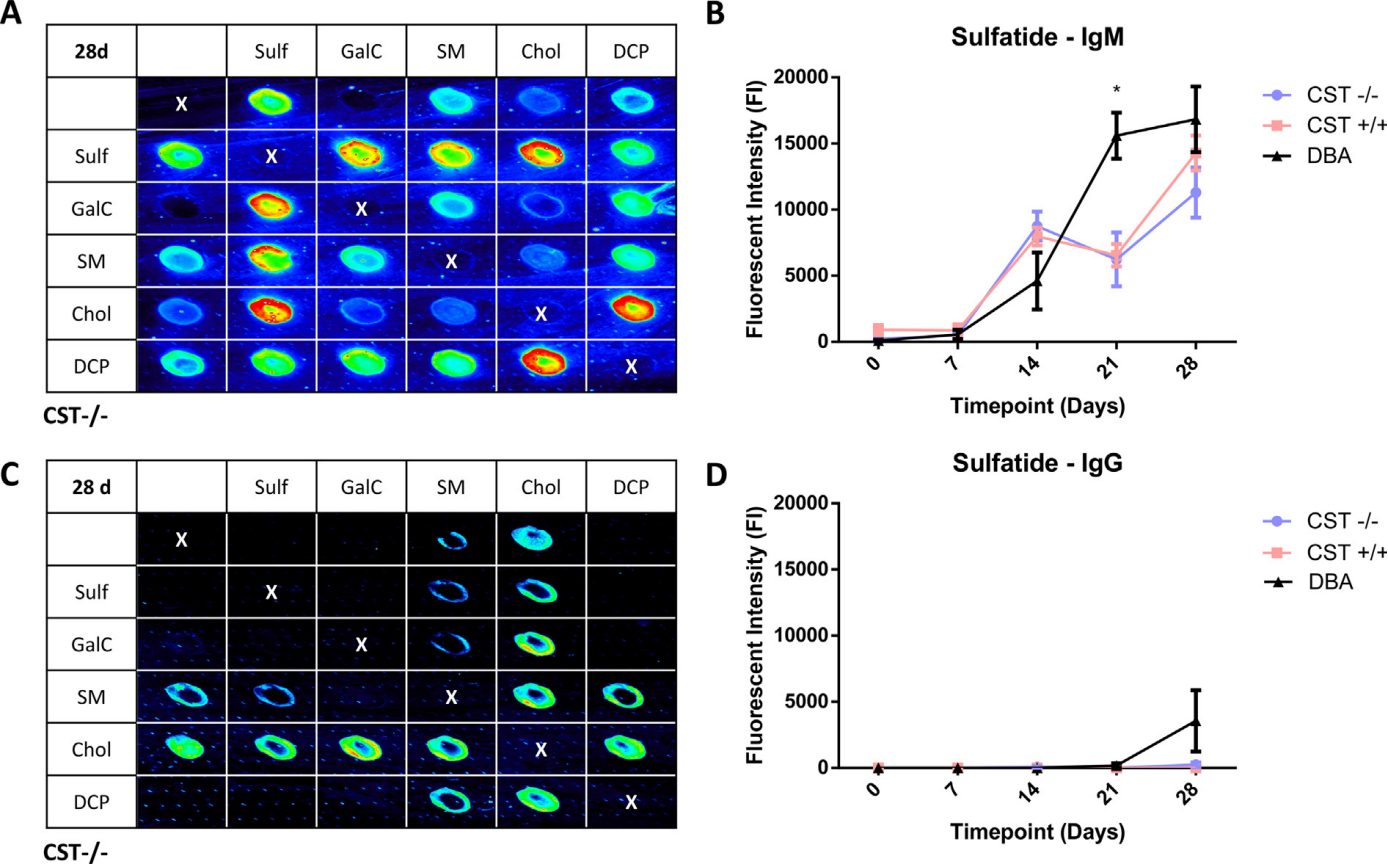
Mice produce anti-sulfatide antibodies in response to immunisations with sulfatide containing liposomes. CST^−/−^(n = 6), CST^+/+^ (n = 3) and DBA mice (n = 3) were immunised with liposomes comprised of sulfatide, SM, Chol and DCP. Combinatorial glycoarray blots from the terminal bleed sera and plots of the single sulfatide reactivity (measured by fluorescent intensity on array) in sera over time indicate the IgM and IgG antibody responses. (A) IgM antibodies against the liposome components could be detected in sera from all genotypes, particularly against sulfatide and sulfatide-associated complexes. A representative blot from CST^−/−^ mouse sera is shown. (B) The levels of anti-sulfatide IgM reactivity rose with each consecutive immunisation but there was no significant/measurable difference between the CST^−/−^ and CST^+/+^ sera at any time-point. In contrast, the level of anti-sulfatide antibodies in the DBA mice were significantly higher than the other genotypes on day 21 (two way ANOVA, P < 0.05). (C) IgG antibodies were detected against SM, Chol and associated complexes but negligible reactivity to sulfatide was detected. A representative blot from CST^−/−^ mouse sera is shown. (D) Anti-sulfatide IgG antibodies only appeared in the DBA sera on Day 28. There was no detectable anti-sulfatide IgG antibodies in the sera of either the CST^−/−^ or CST^+/+^ mice. Array areas spotted with vehicle only are marked with an X. (For interpretation of the references to colour in this figure legend, the reader is referred to the web version of this article.)

CST^+/+^, CST^−/−^ and DBA mice responded similarly to immunisations with sulfatide containing liposomes. In order to fully delineate antibody binding to sulfatide in the presence and context of accessory binding lipids, sera and monoclonal antibodies were screened using a combinatorial glycoarrray approach. Combinatorial glycoarrays from serum taken at day 28 post immunisation indicated that IgM antibodies were generated to all membrane lipids comprised in the liposomes (sulfatide, sphingomyelin, cholesterol and DCP) and their complexes; fluorescence intensity represents the binding signals (Fig. 1A). Antibodies to galactocerebroside were not produced. All genotypes and strains studied generated anti-sulfatide IgM antibodies, which could be detected in their sera from 14 days post-immunisation (Fig. 1B). DBA mice showed a rise in anti-sulfatide IgM antibody response with each subsequent immunisation and on day 21 the antibody level in these mice was significantly greater than the other genotypes (two-way ANOVA, P < 0.05). All genotypes reached a peak serum response at day 28, which showed no significant difference among groups.

In the glycoarray assay, sera showed a weak IgG response to SM, cholesterol and associated complexes at day 28 in CST^−/−^ and CST^+/+^ mice, but no anti-sulfatide IgG antibody response was detected in the serum of either genotype at any time-point (Fig. 1C). In contrast, low levels of anti-sulfatide IgG antibodies were detectable in DBA mice by day 28 (Fig. 1D).

Spleens were harvested from the mice that produced the strongest IgM antibody responses and fused with a myeloma cell line to create hybridomas. Despite the serological data indicating otherwise for CST^−/−^ and CST^+/+^ mice, both anti-sulfatide IgM and IgG antibodies were detected in the supernatant from these hybridomas when screened by glycoarray. The cell lines were expanded and cloned repeatedly to isolate five anti-sulfatide monoclonal antibodies, four of the IgM class (GAME-M2, GAME-M5, GAME-M6, GAME-M7) and one IgG class (GAME-G3, IgG3 subclass), confirmed by screening for sulfatide reactivity (Table 1).

**Table 1:** Binding specificity of anti-sulfatide antibodies as determined by lipid microarray. The antibodies were screened at 10 μg/ml for IgM and 1 μg/ml for IgG on lipid microarray. Values indicate the median fluorescent intensity (MFI) of the antibodies to sulfatide.

Group	mAb	Immunogen	Genotype	Isotype	Sulfatide MFI
A	GAME-M5	Sulfatide liposomes	CST+/+	IgM	5279
A	GAME-G3	Sulfatide liposomes	CST+/+	IgG3	5029
B	GAME-M2	Sulfatide liposomes	CST+/+	IgM	43341
B	GAME-M6	WLE liposomes	CST+/+	IgM	22901
B	GAME-M7	WLE liposomes	CST+/+	IgM	9322
–	O4	Homogenate of Bovine White Matter		IgM	11325

### 3.2 Monoclonal anti-sulfatide antibodies bind to sulfatide and sulfatide-associated complexes

The binding intensities of the antibodies to various lipid antigens were determined by lipid microarray. All five antibodies bound strongly to sulfatide (Table 1). Binding was not detected to any of the following single lipid antigens that were screened: GM1, GM3, GD1a, GD1b, GT1a, GQ1b, GD3, cholesterol, DCP, SM, DPPC, PC, SGPG, and LM1 (data not shown).

**Fig. 2:**
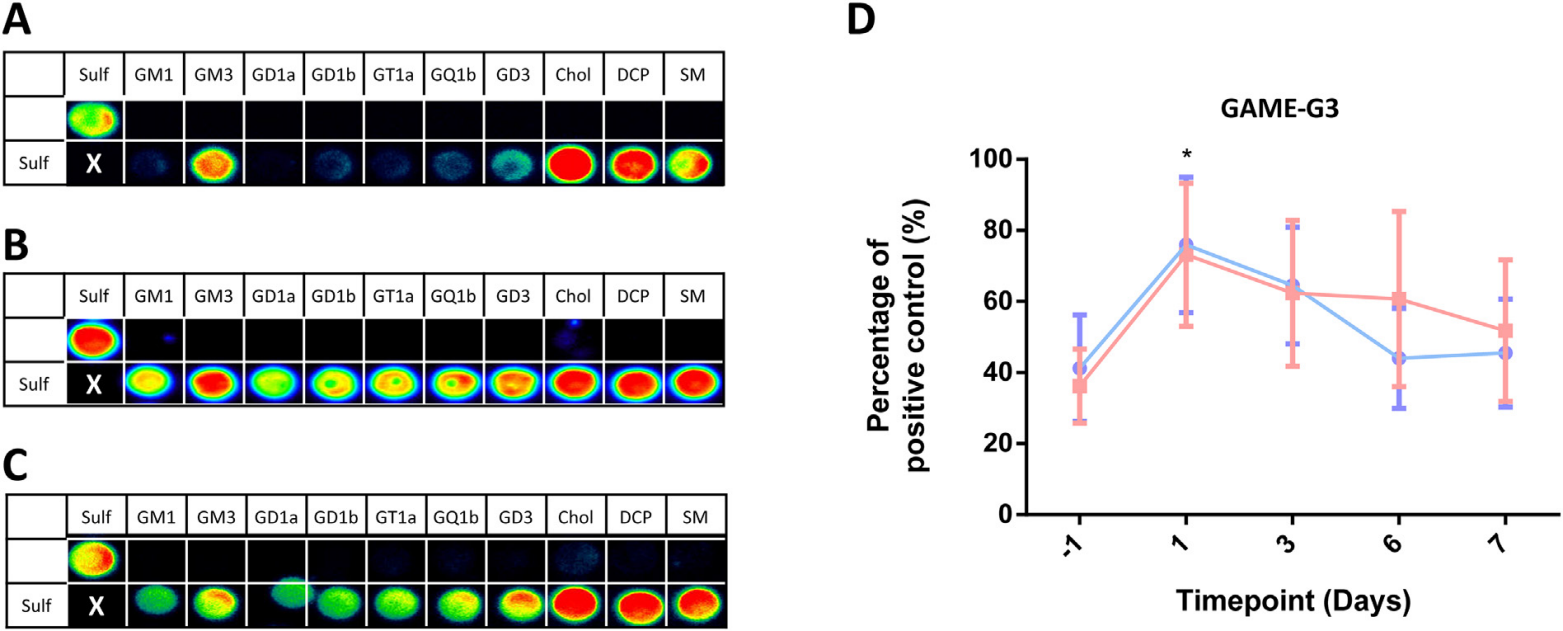
Monoclonal anti-sulfatide antibodies bind to sulfatide and associated complexes on lipid microarray. Newly generated monoclonal anti-sulfatide antibodies were screened against sulfatide antigen printed on a lipid microarray. The IgM antibodies were screened at 10 μg/ml and the IgG antibodies were screened at 1 μg/ml. Their binding patterns fell into two broad categories. (A) Group A comprises GAME-M5 and GAME-G3 antibodies that bind exclusively to sulfatide and sulfatide complexes that do not contain complex gangliosides at the concentrations used. (B) Group B comprises GAME-M2, GAME-M6 and GAME-M7 antibodies that bind strongly to sulfatide and are not affected by associated complexes. (C) O4 was screened for comparison purposes, and showed binding to sulfatide and associated complexes in a similar pattern as the Group B antibodies. (D) CST^−/−^ (n = 6) and CST^+/+^ (n = 6) mice were passively immunised with 250 μg of GAME-G3 antibody. Anti-sulfatide antibody levels were significantly higher following immunisation on day 1 compared to day −1 (baseline) for both genotypes. Clearance from the circulation does not differ between the two genotypes, implying that clearance is not due to receptor-dependent uptake as has been seen for antibodies against gangliosides. Results represent two independent experiments each with 3 mice per group. * Significance at day 1 *versus* day −1, two-way ANOVA with Sidak's multiple comparison test, p < 0.05. Array areas spotted with vehicle only are marked with an X.

In addition to determining the ability of the antibodies to bind single lipid antigens, glycoarray was used to establish how the antibodies bound to various lipids in complex with sulfatide. In general, the antibodies fell into two broad categories, herein termed Groups A and B. Group A (GAME-M5 and GAME-G3) bound to sulfatide and some sulfatide complexes, but were unable to bind sulfatide when presented with complex gangliosides (Fig. 2A). Group B antibodies bound all sulfatide-associated complexes regardless of interaction with any other lipids or glycolipids tested, including complex gangliosides (GAME-M2, GAME-M6 and GAME-M7) (Fig. 2B). O4 was found to be most similar to the group B antibodies as it bound to all sulfatide associated complexes at this antibody concentration (Fig. 2C).

Serologically, CST^−/−^ mice responded to sulfatide immunisation similarly to CST^+/+^ mice. Interestingly, hybridoma screening revealed the presence of IgG antibodies, which were not detected in mouse serum. We therefore considered that any circulating anti-sulfatide antibodies might be actively depleted from the circulation through a receptor-dependent endocytosis clearance mechanism, as previously observed for anti-ganglioside antibodies (Cunningham et al., 2016). To investigate this, CST^+/+^ and CST^−/−^ mice were passively immunised with 250 μg/ml of GAME-G3 and bled at days 1, 3, 6 and 7 to monitor serum levels of anti-sulfatide antibodies (Fig. 2D). Both genotypes had significantly higher levels of anti-sulfatide antibodies on day 1 following passive delivery of GAME-G3 compared to baseline, as would be expected, and were not significantly different to each other (two way ANOVA, P < 0.05). Over the following days, there was no significant difference in antibody levels between the genotypes at any time-point. This indicated that the antibodies were not being actively cleared from the circulation through endocytosis in an antigen-dependent manner.

### 3.3 Anti-sulfatide antibody binding to biological membranes is determined by physiological condition

**Fig. 3:**
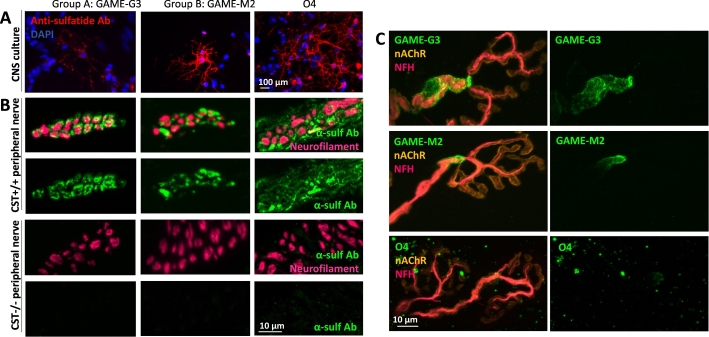
Monoclonal anti-sulfatide antibodies bind to myelinating CNS cultures, peripheral nerve sections and *ex vivo* nerve-muscle preparations. (A) Representative images indicate the relative binding patterns of the Group A, Group B and O4 anti-sulfatide antibodies to myelinating CNS cultures. All antibodies (red) were all able to bind to the oligodendrocytes in CNS cultures to varying degrees; the group B antibodies and O4 displayed the strongest signal. (B) Representative images indicate the relative binding patterns of the anti-sulfatide antibodies (green) to peripheral nerves identified by axonal neurofilament labelling (magenta) in transverse sections. Group A and Group B antibodies bound well to the transverse nerve bundles from CST^+/+^ mice, O4 exhibited more diffuse binding. None of the antibodies bound to tissue from CST^−/−^ mice confirming that they selectively bind to sulfatide. (C) The Group A (GAME-G3) and group B (GAME-M2) anti-sulfatide antibodies (green) bound along the myelin sheath encircling neurofilament labelled axons (magenta) and intensely at the terminal heminode paranodal loops in the mouse distal motor nerve when applied topically to an *ex vivo* nerve-muscle preparation. In contrast, O4 was unable to bind or bound comparatively weakly to the distal nerve myelin. (For interpretation of the references to colour in this figure legend, the reader is referred to the web version of this article.)

Anti-sulfatide mAbs were next probed against cells and tissues to determine if they were capable of binding to sulfatide as it is physiologically displayed in living biological membranes. Binding studies were first carried out by probing the antibodies against myelinating CNS cultures, in which oligodendrocytes are known to express high levels of sulfatide and bind O4 (Mirsky et al., 1990; Reynolds and Hardy, 1997). Within both groups, mAbs demonstrated similar binding patterns, one antibody from each group being illustrated (Fig. 3A). Whilst all antibodies in Groups A and B bound to oligodendrocytes in myelinating cultures, group B antibodies and O4 bound more strongly than those in group A. Based upon the glycoarray screening it is thus possible that sulfatide on oligodendrocyte membranes may cis-interact in the plane of the plasma membrane with neighbouring gangliosides or other glycolipids, thereby preventing access to the epitope on sulfatide that Group A antibodies target. Such an interaction would result in attenuated binding.

Subsequently, the mAbs were probed against peripheral nerve transverse sections from both CST^+/+^ and CST^−/−^ mice to determine how they interacted with sulfatide in mature myelinated peripheral nerve tissue (Fig. 3B). All the antibodies bound strongly to the transverse nerve bundles in the CST^+/+^ tissue, whereas no binding was detected in the CST^−/−^ tissue, confirming that the antibodies were specifically binding to sulfatide. Binding encircled the axonal neurofilament staining in a halo pattern, suggesting labelling of the sulfatide-enriched myelin. Additional extra-myelinic staining of structures was present with the O4 mAb, albeit weakly in the CST^−/−^ tissue, suggesting non-specific binding. To determine whether the mAbs were able to bind physiologically intact living nerve membranes, we first used live *ex vivo* triangularis sterni (TS) nerve muscle preparations that contain distal myelinated motor nerve fibres (Fig. 3C). Both group A and group B mAbs bound along the myelin internode and strongly at the paranodal loops of the terminal heminode. In comparative contrast, binding with O4 was weaker, patchy in distribution, or undetectable, despite having a similar binding profile to Group B antibodies. Binding of all mAbs was most prominent distally, due to available access to this site and did not advance further proximally, presumably due to limitation on access afforded by the blood-nerve barrier (BNB).

### 3.4 Anti-sulfatide antibody binding to mouse peripheral nerve following passive immunisation *in vivo*

**Fig. 4:**
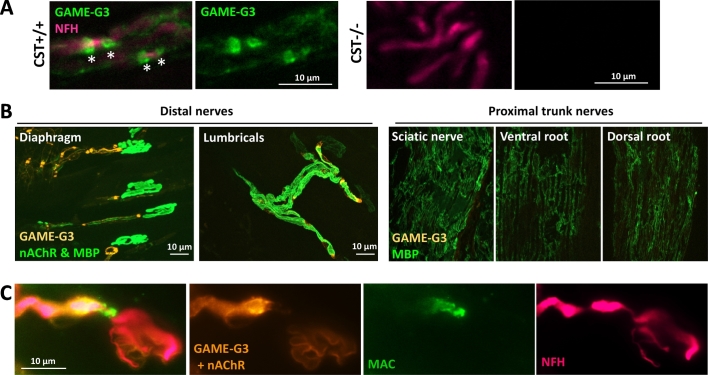
– Anti-sulfatide antibodies are capable of binding *in vivo* and fixing complement in *ex vivo* nerve-muscle preparations. (A) Binding along the myelin and at the paranodal loops (green, *) was observed in the peripheral nerves (magenta) of CST^+/+^ mice after *in vivo* delivery of 1 mg GAME-G3 i.p. the previous day. No binding was observed in CST^−/−^ mice demonstrating the specificity of the antibody to sulfatide. (B) 1 mg GAME-G3 was injected i.p. to compare and study access at distal and proximal sites. 24 h later binding could be observed in the distal nerves (whole-mount diaphragm and lumbricals), while binding was absent from proximal nerve trunks (sciatic nerve, spinal root sections). C) GAME-G3 antibody binding (orange) in *ex vivo* TS muscle was capable of activating the complement cascade in distal motor nerves (magenta), culminating in the deposition of the membrane attack complex (MAC; green). (For interpretation of the references to colour in this figure legend, the reader is referred to the web version of this article.)

The ability of the anti-sulfatide antibodies to specifically bind sulfatide-containing neural membranes *in vivo* was assessed by passively immunising CST^+/+^ and CST^−/−^ mice with the anti-sulfatide IgG mAb, GAME-G3 (1 mg total dose per mouse, delivered IP). When intramuscular nerve fibres were examined for the presence of mAb deposits, prominent binding was observed in paranodal regions and at the terminal hemi-node. In contrast to the situation in transverse frozen sections, where internodal myelin staining was prominent, internodal myelin was weakly labelled compared with paranodal Schwann cell membranes. Figs. 4A & B demonstrate prominent GAME-G3 binding at paranodal loops (asterisks) and weaker staining along the internodal myelin in small intramuscular peripheral nerve bundles from CST^+/+^ mice. As expected, CST^−/−^ mice demonstrated no binding. When examining more proximal sites such as the sciatic nerve and spinal roots we detected no antibody binding (Fig. 4B), after up to 5 days of exposure *in vivo*. To assess the functional capacity of bound antibody, we exposed mouse distal nerves to GAME-G3 and a source of human complement in a live *ex vivo* nerve-muscle preparation as above and probed the tissue for evidence of complement activation. GAME-G3 bound to the distal nerves and activated the complement cascade, culminating in the deposition of membrane attack complex (MAC) pores at distal myelin and nodes of Ranvier (Fig. 4C).

## 4 Discussion

Monoclonal antibodies (mAbs) of known antigen specificity and tissue binding capability are an invaluable resource to study antigen localisation and function, and the immunopathogenesis of autoantibody-mediated autoimmune disease. A particular feature of anti-glycolipid mAbs is the diversity of binding patterns exhibited by antibodies to a single glycan epitope, according to the topographical orientation of the glycan in the plane of the plasma membrane. This diversity confounds antigen localisation studies, which for sulfatide are historically mostly defined by the bindings patterns of the O4 mAb (Sommer and Schachner, 1981). Differential binding of different anti-sulfatide antibodies to sulfatide-containing membrane domains will also affect both the vulnerability of tissues to immune-mediated attack and the pathogenicity of the antibodies that arise in various neuropathies. These immunopathological features will not be deducible from interrogating solid phase immunoassays such as ELISA or glycoarray in which purified antigens are adhered to artificial surfaces prior to probing with antibody. For sulfatide and other glycolipids, the antibody-binding epitopes may be obscured or exposed depending upon the local microenvironment (Galban-Horcajo et al., 2014; Greenshields et al., 2009; Nobile-Orazio et al., 2014). Studying these subtle nuances in the context of sulfatide would greatly benefit from the development of a greater selection of monoclonal antibodies for neuropathy pathogenesis studies than O4 provides. Understanding the characteristics of these varied binding patterns will also allow us to determine the patterns of sulfatide reactivity that are found in human neuropathy and other sera, and thereby improve the screening conditions for identifying immunopathologically relevant human antibodies.

The mAb O4 is most commonly used to study and localise sulfatide. Whilst O4 exposure has been shown to produce demyelination of CNS tissue both *in vitro* and *in vivo* (Rosenbluth and Moon, 2003; Rosenbluth et al., 2003), its use in studying sulfatide mediated peripheral nerve pathology has proven difficult. In our previous studies, we have been unable to demonstrate anatomically interpretable and specific binding of O4 to live peripheral nerve preparations, despite it being similar to Group B antibodies. The explanation for this at the structural level is unknown. As sulfatide is known to be expressed in peripheral nerve myelin membranes, the absence of binding suggests that O4 is unable to access the sulfatide epitope in Schwann cell membranes. Thus it may not represent the heterogeneity of the anti-sulfatide antibody populations that arise in humans with either CNS or PNS demyelinating diseases, or in immunised animals (Brennan et al., 2011).

We sought to address this by generating and characterising a larger series of anti-sulfatide mAbs. This was achieved by B-cell cloning from CST^−/−^, CST^+/+^ and DBA mice immunised with sulfatide and WLE liposomes. DBA mice produced higher levels of anti-sulfatide antibodies compared with CST^+/+^ and CST ^−/−^ mice the latter being a C57BL/6J background. Previous success in using complex ganglioside deficient mice to generate anti-ganglioside antibodies (Bowes et al., 2002; Lunn et al., 2000) led us to select the sulfatide deficient CST^−/−^ mouse for immunisations. We expected that these mice would be immunologically naïve, due to a lack of exposure to endogenous sulfatide, and would therefore form a more robust immune response compared to sulfatide-replete wild type mice. However, CST^−/−^ mice did not produce anti-sulfatide antibodies in greater amounts than their wild type counterparts, indicating that antigen naïvety does not play an obvious role in regulating immune tolerance to sulfatide. The role of sulfatide-restricted NK T cell activation by exogenous sulfatide administration was not explored but is a possible confounding factor (Rhost et al., 2014).

The panel of anti-sulfatide mAbs were shown by combinatorial glycoarray to have two binding patterns and were also distinct in some binding capacities from the prototypic O4 mAb. The combinatorial element of the glycoarray permitted the examination of how heteromeric complexes of lipids affected antibody binding that can then be compared with binding patterns in physiologically intact membranes. In the presence of gangliosides as heteromeric partners, Group A mAbs bound to sulfatide less well than Group B mAbs in solid-phase assays. This lower binding signal was also reflected in the CNS cultures. As certain gangliosides, particularly GD3 (Reynolds and Wilkin, 1988), are enriched in these myelinating cultures, it is possible that the epitope of sulfatide favoured by Group A antibodies is not optimally exposed, limiting the binding ability of these antibodies. This same inhibition, however, was not observed in peripheral nerve sections or *ex vivo* preparations, suggesting that sulfatide was presented differently in these tissues.

Whilst we confirmed that O4 mAb also bound sulfatide in solid-phase arrays, myelinating culture and peripheral nerve sections, the pattern was much more diffuse compared to the GAME mAbs that localised quite precisely to myelin and paranodal membranes. In particular, O4 mAb was rarely detected along the myelin in the distal motor nerve of *ex vivo* preparations, in striking contrast to the GAME mAbs. This suggests that the newly generated anti-sulfatide GAME series antibodies bind to slightly different epitope configurations or density arrangements compared with O4 mAb, despite O4 mAb being a Group B antibody. Antibody binding along distal nerves was very strong 24 h after *in vivo* delivery into CST^+/+^ mice and not CST^−/−^ demonstrating the specificity of the antibody to sulfatide. Binding was observed on the distal nerves of the diaphragm and lumbrical muscles, suggesting the antibody was capable of entering the systemic circulation. However, the antibody appeared to be occluded from trunk nerves, likely owing to the protection afforded by the BNB. It is possible that higher doses or manipulation of the BNB permeability could allow access of antibody and will be worth further investigation.

An important feature of the GAME anti-sulfatide mAbs was their ability to activate the complement cascade at the distal motor nerve. Whether this produced a pathological lesion in the myelin and Schwann cell membranes remains to be determined in further studies. Although O4 is also capable of activating complement, its inability to bind this peripheral site gives the GAME antibodies an advantage in studying murine models of human disease, as demyelination of the nerve is observed with certain anti-sulfatide antibody-associated neuropathies and demyelination has been associated with complement activity (Rosenbluth and Moon, 2003; Storch and Lassmann, 1997). Complement may not be the only effector of myelin disruption in demyelinating diseases (Genain et al., 1999) and other complement-independent injury routes for these antibodies should be included in future characterisations.

Due to the discrepancies in antibody presence between results from serology and high-throughput screening of hybridoma supernatant, we also investigated whether anti-sulfatide antibodies were removed from the circulation via endocytic uptake, using GAME-G3 as a representative antibody. In contrast to a previous study using anti-ganglioside antibodies (Cunningham et al., 2016), comparisons of CST^−/−^ and CST^+/+^ indicated that the antibodies were not removed from the circulation by sulfatide-mediated endocytosis. Despite the mechanism of antibody clearance remaining unknown, the discrepancy between detectable levels of IgG in the serum and in hybridoma supernatant highlights the importance that serum levels of antibody do not represent the repertoire of antibodies being produced (Cunningham et al., 2016).

In conclusion, we have generated and performed preliminary characterisation of a series of new anti-sulfatide antibodies and demonstrated that they have a range of binding patterns that are distinct from those of the most commonly studied anti-sulfatide antibody, O4. By studying a more diverse range of antibodies, we will build upon the existing knowledge of binding behaviours, which will ultimately aid us in modelling and understanding their roles in autoimmune neuropathies.

## Funding sources

G.R.M. was funded by a Guillain-Barré & Associated Inflammatory Neuropathies (GAIN) charity studentship. Experimental studies were supported by Wellcome Trust grants 202789/Z/16/Z and 092805/Z/10/Z.

## Conflicts of interest

None.
